# Response to the letter ‘Field classification of publications in Dimensions: a first case study testing its reliability and validity’

**DOI:** 10.1007/s11192-018-2854-z

**Published:** 2018-07-27

**Authors:** Christian Herzog, Brian Kierkegaard Lunn

**Affiliations:** Digital Science, 90 York Way, London, N1 9AG UK

**Keywords:** Bibliometrics, Dimensions, Lutz Bornmann, Digital Science, Research classification, Machine learning

## Abstract

With Dimensions, Digital Science provides the research community a new approach on research related information, bringing formerly siloed content types such as grants, patents, clinical trials with publications and citations together, making it as openly available as possible (see app.dimensions.ai). Due to the different content types, (controversial) journal based classifications were not an option since it would not allow to categorise grants etc. Hence Digital Science opted for applying a categorisation approach using machine learning and based on the content of the documents and well established classification systems for which a training set was available. The implementation at launch was a first step and requires to be improved—although we observe a reliability comparably to manual coding for grants, the implementation at launch comes with some shortcomings as observed by Bornmann (2018), mostly due to challenges with the training set coverage. To overcome the shortcomings of the initial categorization approach we implemented an improvement process with the research community and Lutz Bornmann’s analysis presented a great opportunity to provide more transparency and insights in the ongoing improvements.

## Summary of the main remarks to the letter

Based on the collaboration in the development partner context with Lutz Bornmann we discussed how the largest transparency possible can be created about the approach for the automatic classification, its limitations and the current and planned efforts improve the classification schema and results. We decided to try the format of a ‘letter and response as a dialogue’—where the findings are coupled with backgrounds, comments and steps to address unsatisfying results—together with the research community.

Bornmann (2018) analysed the classification of publications assigned to his profile in Dimensions (see https://app.dimensions.ai/discover/publication?and_facet_researcher=ur.01041121777.72). He observed that most publications has been classified in a suboptimal way. While the sample size of about 260 compared to 94 million publications is quite small and the selection of only one researcher profile is not representative, the findings are relevant, pointing towards weaknesses not necessarily inherent to the approach chosen, but the current challenges of the implementation. These can be summarised mainly in the two aspects:

### Choice of the classification systems for the free version of Dimensions

With the Fields of Research (FOR) from the Australian and New Zealand Standard Research Classification (ANZSRC) we have optes for a general classification system, covering the different areas of science as broadly as possible (Australian and New Zealand Standard Research Classification 2008) which is implemented alongside other classification systems. The decision was also driven by the requirement that a training set is available to implement the machine learning based classification approach. The field of ‘research evaluation, including scientometrics, informetrics, bibliometrics, and altmetrics’ is not covered specifically on the 4 digit layer of the FOR coding system—and also not on the 6 digit level, which we decided not to implement due to too few records in the training set per code. The closest code is *080705 Informetrics* (Group 0807 Library and Information Studies 2008), but even this would only cover parts of the publications in questions.

The trade-off between a general system and one which covers specific aspects like ‘science of science’ in particular will be always challenging, Lutz Bornmann has highlighted here the impact of Dimensions leaning towards a more general approach—with implications for field weighted indicators—possible approaches (such as improvements to the current schema or subject specific classifications will be discussed later).

### General size and quality of the training sets per category

Another limitation about which we had been forthcoming at launch of Dimensions is the size and quality of the training set which was available to us train the machine learning model. For the FOR system, the training set consisted at the start of funded grants from the Australian Research Council (ARC) and the National Health and Medical Research Council (NHMRC). The fact that funded grants and not publications are used is already creating a certain bias in addition to the structure and granularity mentioned in the previous paragraph. Since the training sets can be improved and augmented, we have planned from the start for an annual refresh (see Bode et al. 2018, p. 5)—the current status and planned actions are also discussed later.

## Background to the categorisation systems and process in Dimensions

### Moving past journal level classifications

The decision to not implement a journal level categorisation system but an approach, which categorises documents based on the title and abstract (and only the records where both title and abstract are available) was triggered since Dimensions focuses on linking different content types and grants or patents do not have a journal attribute. The approach of using journal level classifications has been well studied (e.g. Waltman and van Eck 2018; Glänzel et al. 1999) and the shortcomings (especially in the area of interdisciplinary journals) are clearly described and mentioned by Bornmann (2018).

With the approach using machine learning to automatically assign categories based on an established classification system and a training set we put an infrastructure in place to implement many classification systems in parallel and to start the process of a continuous development and improvement of the category systems. The different versions of Dimensions currently have different classification systems—depending on the user groups, focus and use cases:

- *Free version of Dimensions and Dimensions Plus* general discovery is the main use case, FOR has been chosen as the classification system due to the high level coverage of all research areas.
- *Dimensions Analytics* funders and research administration are the main users of this Dimensions variant—and based on client request other classifications have been integrated, covering specific areas of research, but using the same machine learning approach and infrastructure. All of these are also implementations of existing classification systems—such as the ‘Research, Condition, and Disease Categorization (RCDC)’ from the NIH, the ‘Health Research Classification System (HRCS)’ from the UK or private implementations of disease specific classification system.

While we are convinced that moving past metadata driven classifications is the right path forward, since the general trend is to look across content types—it is also clear that such a new approach will require iterations, will never be solved satisfactory with a single universal classification system and will require a larger effort involving the research community, especially if the classifications are used for field normalised indicators.

In general, we designed Dimensions to be ready to host 1 − *n* classification systems using the same technological approach which allows to implement multiple classification systems at the same time—to take the need for a ‘universal multi-purpose tool’ away and have use case specific targeted classifications applied in parallel. And at the same time this allows as well to have iterations of one classification system which improves over time.

## Different approaches to address the shortcomings of the current FOR implementation

While implementing the initial version of Dimensions with the FOR categorisation approach we started to prepare how the continuous improvement can be realised—and settled on four approaches with different levels of complexity:

1. Improvement of the training sets working within the structure of the FOR categorisation system—to strengthen the training set for challenging categories.
2. Expand the implementation of the FOR system to the 6-digit level to add more granularity: before investing the significant effort on basically creating the training sets a detailed analysis of the value created needs to be carried out. The current set of categories on the 4 digit level includes *n* = 157 ‘groups’ in the FOR nomenclature, while the 6-digit level has already 1238 fields.
3. Implementation of categories beyond the FOR system: new subcategories can be added to a (then Dimensions specific variant) of the FOR classification system.
4. Crowd sourcing of feedback on existing categorisation on an article-level.

## Improvement of the training sets: work in progress

Over the past months, we have done a thorough analysis of the training sets on a category level to identify categories where an improvement fort the training set can lead to improved results:

We developed a workflow and tool based active learning principles to improve the categories with a consistent approach which allows us to involve subject matter experts for the field of the category in a guided process.

This allowed us to test our workflow in the area of Lutz Bornmann’s research activities due to internal subject matter expertise in that area—which allowed us to see whether the improved category would correctly classify them in *0807 Library and Information Studies*, the closest category in the FOR system as mentioned above, and thereby get a first glance of the reliability and validity of the improved category 0807.

After running a first test of the workflow we observed a significant improvement in assigning out of the 201 eligible publications (61 do not have an abstract in Dimensions and are therefore not categorised) more than 45% were classified in the category *0807 Library and Information Studies*—compared to 0 in the currently deployed implementation. Though encouraging results for a first analysis, this testifies to the fact that this is ongoing work, with room for improvement—both working on the process and the manual analysis of the set since scientometrics research can be applied and focused on specific areas, making it more challenging to detect.

In addition to improving the training set it is also possible to adjust the matching algorithm e.g., with respect to weights between recall and precision. By adjusting the weight towards a high recall and include publications without abstracts, 90% of the 262 publications are assigned the improved 0807 category. Weighting recall over precision have the natural risk of assigning categories to too many publications, and hence such adjustment between recall and precision needs to be examined in detail across the whole corpus of 90 million publications before being implemented. This said, this too is an encouraging results, which we will be examining further in the coming future.

Analysing in more detail the publications not categorised with 0807 will also be our focus in the coming months as scrutiny of the non-matched publications is a natural way to learn how to further improve this category. That said, no matter how much we improve training set and adjust algorithm it will never be possible to categorise all 262 publications in this category.

Going forward, this process will be applied to other challenging categories (such as physics categories) involving subject matter experts.

## Summary

The implementation of a content based classification will always be work in progress and we encourage more feedback like the findings from Lutz Bornmann since it draws attention to the work ahead. And the improvement of the training sets for certain categories seems to achieve a more precise classification in fewer categories.

The most promising option seems to be to work on a specific ‘mini’ classification system for the ‘field of research evaluation, including scientometrics, informetrics, bibliometrics, and altmetrics’. It could be an interesting exercise to involve multiple subject matter experts—especially given the ‘applied’ and interdisciplinary nature of some of the research activities in these fields. We would be happy to assist in such an exercise and provide Dimensions as a data infrastructure and implementation platform.

For Dimensions, we decided to go for a first version of a generic classification system which limited granularity by implementing an existing and accepted classification system, focusing on discovery support and field weighted indicators as the main use cases, while being technically able to assign it to all content types. But is only a first implementation and we are committed to improve in a transparent process in the coming iterations.

In addition we will continue our efforts to improve the categorisation within the structure of the FOR system by improving the training sets—with the research community and subject matter experts—any input and support is welcome.
